# ﻿Review of the genus *Xanthephialtes* Cameron (Hymenoptera, Ichneumonidae), with re-definition of the genus and description of a new species from Uganda

**DOI:** 10.3897/zookeys.1266.175534

**Published:** 2026-01-05

**Authors:** Emil M. Österman, Tapani Hopkins, Ilari E. Sääksjärvi, Kari M. Kaunisto, Simon van Noort

**Affiliations:** 1 Biodiversity Unit, Zoological Museum, University of Turku, 20014 Turku, Finland University of Turku Turku Finland; 2 Research and Exhibitions Department, South African Museum, Iziko Museums of South Africa, PO Box 61, Cape Town, 8000, South Africa South African Museum, Iziko Museums of South Africa Cape Town South Africa; 3 Department of Biological Sciences, University of Cape Town, Private Bag Rondebosch, 7701, South Africa University of Cape Town Cape Town South Africa

**Keywords:** Africa, Darwin wasp, Kibale National Park, morphological variation, taxonomy, Uganda Malaise trapping 2014–2015

## Abstract

*Xanthephialtes* Cameron, 1906 (Hymenoptera, Ichneumonidae, Pimplinae) is a small Afrotropical Darwin wasp genus with only two previously known species, for which we provide images of the holotypes. Here, we describe a new species of the genus, *X. iida***sp. nov.**, which is also the first record of the genus in Uganda. We provide a re-definition of the genus and a key to species along with notes on the morphological variation and mimicry of *X. schoutedeni* Benoit, 1954. Online Lucid identification keys and images of all the species treated herein are available at http://www.waspweb.org.

## ﻿Introduction

*Xanthephialtes* Cameron, 1906 is a small genus within the tribe Ephialtini of the Pimplinae subfamily of Darwin wasps (Hymenoptera, Ichneumonidae). Until now, the genus included only two known species: *X. oculatus* (Brullé, 1846) and *X. schoutedeni* Benoit, 1954 (Dal Pos et al. 2023; Bánki et al. 2025). Previous records of the genus are from Burundi, Guinea, the Democratic Republic of Congo, and South Africa (Dal Pos et al. 2023), suggesting a widespread distribution across continental Africa. Unfortunately, there are no known host records for species of this genus.

**Figures 1–5.**
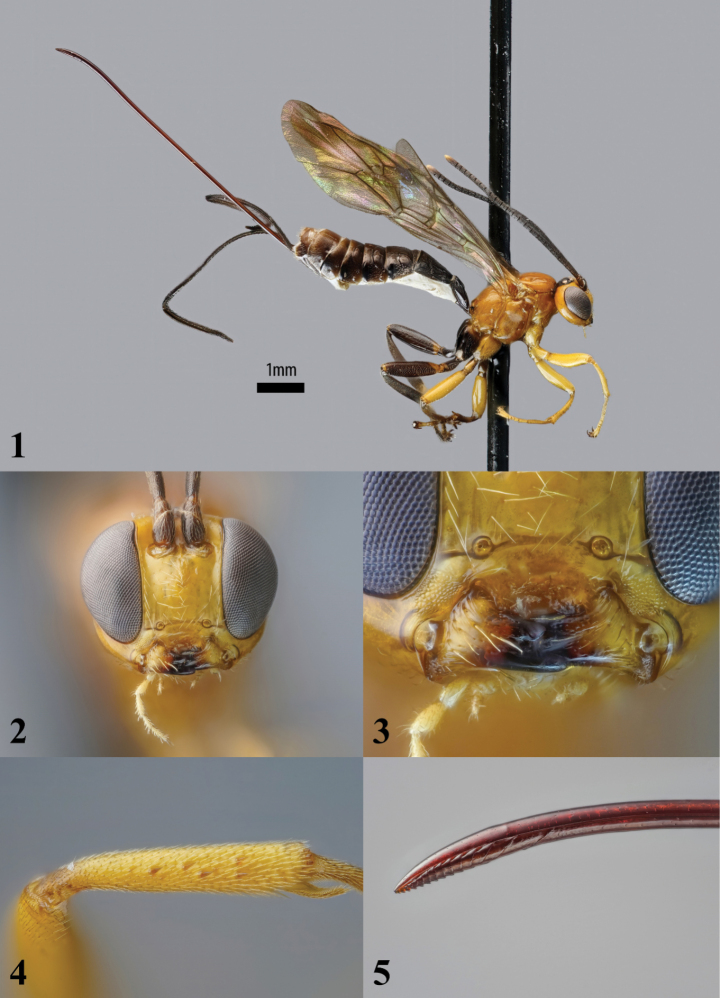
*Xanthephialtes iida* sp. nov., holotype female (ZMUThttp://mus.utu.fi/ZMUT.3563). **1.** Habitus, lateral view; **2.** Head, anterior view; **3.** Mandibles, anterior view; **4.** Flattened bristles on foretibia, antiaxial view; **5.** Ovipositor apex, lateral view.

*Xanthephialtes* can easily be distinguished from other pimpline genera (particularly *Dolichomitus* Smith, 1877, to which it bears some resemblance) by the combination of the following characteristics: ventral mandibular tooth conspicuously longer than dorsal tooth (Fig. 3); flattened bristles on foretibia short and stout (Fig. 4); mesosoma mostly impunctate, with weak punctures in some areas; first tergite depressed, almost impunctate, and lacking latero-median carinae (modified from Townes 1969). The ovipositor is relatively long and apically downcurved (Figs 1, 5), similar to that of some species of *Dolichomitus*. However, as *Dolichomitus* seems to be absent from Africa, there is little risk of confusion with other pimpline genera.

In this article, we describe a new species, *Xanthephialtes iida* sp. nov., from Uganda, provide a re-definition of the genus and images of the holotypes along with a key to species, and include notes on the morphological variation and mimicry of *X. schoutedeni*. This article is the first in a series of forthcoming publications describing new pimpline species collected in an extensive one-year sampling in Kibale (reported in Österman et al. 2025a).

## ﻿Methods

The Ugandan *Xanthephialtes* specimens studied here were separated from samples collected with 34 Malaise traps that operated for one year (2014–2015) in Kibale National Park, Uganda. The traps were placed in various habitats that varied across a successional gradient from primary forest to farmland. The sampling effort totaled 382.4 trap months, of which 22.6 trap months were damaged samples or otherwise provided at most a partial catch of Pimplinae. The study area and sampling campaign are described in greater detail in Hopkins et al. (2019a, 2019b).

We photographed the Ugandan specimens, including the new holotype, at the Zoological Museum, Turku (ZMUT) using a Sony Alpha 9 Mark II camera body mounted on a macro rail, which enabled us to control and incrementally move the camera between shots. Photos were captured using an extension tube, a relay lens, and Mitutoyo Plan Apo objectives with magnifications ranging from 2.5× to 5×. We captured multiple images at successive focal depths and combined them using the software Helicon Focus v. 7.7.5 (Helicon Soft Ltd.) to produce composite layer images with extended depth of field. We carried out final image adjustments in Adobe Photoshop CC to ensure accurate representation of the specimen’s morphological features. Observation at ZMUT were made using Olympus SZ61 and SZX16 stereomicroscopes. Morphological terminology follows Gauld (1991) with minor modifications: dorsal/ventral instead of upper/lower when referring to the mandibular teeth and ovipositor valves.

Images of the other holotype specimens were captured in 2003 with a Nikon SLE2, 105 mm macro lens, and 35 mm slide film. Images acquired at the South African Museum, Cape Town (SAMC) used a Leica LAS 4.9 imaging system, comprising a Leica® Z16 microscope (using either a 2× or 5× objective) with a Leica DFC450 Camera and 0.63× video objective attached; diffused lighting was achieved using a Leica LED5000 HDI dome; and the imaging process, using an automated Z-stepper, was managed using the Leica Application Suite v. 4.9 software installed on a desktop computer.

### ﻿Repositories

**MNHN** Muséum national d’Histoire naturelle, Paris, France

**RMCA** Musée Royal de l’Afrique Centrale, Tervuren, Belgium

**SAMC** South African Museum, Iziko Museums of South Africa, Cape Town, South Africa

**ZMUT** Zoological Museum, Biodiversity Unit, University of Turku, Turku, Finland

## ﻿Results

### ﻿Taxonomy

#### ﻿ Xanthephialtes

Taxon classificationAnimaliaHymenopteraIchneumonidae

Cameron, 1906

46738F0C-FA98-5FBF-AB6C-8139B88224AE

##### Type.

*Ephialtes oculatus* Brullé, 1846, by original designation.

##### Re-definition.

Medium-sized to large insects (forewing length 6.5–19 mm) which are generally black and orange-yellow. Clypeus clearly separated from face, impressed, with a deep median notch on apical margin. Mandible basally strongly tapered, apically rather weakly tapered; mandible base oblique, about 28–34° from vertical axis of face, ventral mandibular tooth conspicuously longer than dorsal tooth. Palpal formula 5:4. Occipital carina complete but rather weak. Mesosoma polished with sparse and weak punctures. Epomia weak or absent. Mesoscutum mostly bare, with notauli weakly impressed. Epicnemial carina reaching the level of the middle of the posterior margin of the pronotum. Scutellum convex, without lateral carinae. Propodeum moderately long and rather evenly declivous in profile, with complete pleural carina and posteriorly with short, stub-like lateral longitudinal carina. Submetapleural carina complete. Flattened bristles on foretibia short and stout, numbering about 5–14. Forewing vein *3rs-m* with two bullae, enclosing an obliquely trapezoidal areolet, and *cu-a* opposite base of *Rs&M*. Hindwing with abscissa of *Cu1* between *M* and *cu-a* straight and about long as *cu-a*. First tergite depressed, almost impunctate, and lacks latero-median carinae. Second tergite large, longer than first tergite, with latero-basal, oblique grooves extending to 0.6 length of tergite or more, and with posterior transverse grooves. Tergites 3–5 with smooth latero-median tubercles. Female with ovipositor cylindric, about 4× as long as hind tibia, apically downcurved; ventral valve does not meaningfully enclose the dorsal valve; apex of ventral valve bearing many strong teeth, with a disproportionately large gap between the basal and the second most basal tooth.

#### ﻿ Xanthephialtes iida

Taxon classificationAnimaliaHymenopteraIchneumonidae

Österman sp. nov.

7A8BE57E-47EC-57AC-914B-8C05EDAAC8C8

https://zoobank.org/6A495052-355A-44ED-829C-D2A5CAD25619

Figs 1–5

##### Material examined.

Uganda, Kibale National Park, Kanyawara; Tapani Hopkins leg.; ZMUT: ***Holotype*** • 1 female. Site K13, Malaise trap K13T3; 0.5362°N, 30.3486°E; alt. 1492 m; 15 Dec. 2014–28 Dec. 2014; http://mus.utu.fi/ZMUT.3563. ***Paratype*** • 1 female. Site K31, Malaise trap K31T4; 0.6927°N, 30.3616°E; alt. 1463 m; 17 Dec. 2014–29 Dec. 2014; http://mus.utu.fi/ZMUT.3600. ***Paratype*** • 1 female. Site CC, Malaise trap CCT1; 0.5497°N, 30.3673°E; alt. 1454 m; 13 Jan. 2015–27 Jan. 2015; http://mus.utu.fi/ZMUT.5869. ***Paratype*** • 1 female. Site K31, Malaise trap K31T1; 0.5440°N, 30.3503°E; alt. 1497 m; 9 Apr. 2015–23 Apr. 2015; http://mus.utu.fi/ZMUT.3571. ***Paratype*** • 1 female. Site R98, Malaise trap R98T2; 0.5532°N, 30.3579°E; alt. 1545 m; 8 May 2015–22 May 2015; http://mus.utu.fi/ZMUT.3969. ***Paratype*** • 1 female. Site K30S, Malaise trap K30ST4; 0.5414°N, 30.3755°E; alt. 1421 m; 15 Dec. 2014–26 Dec. 2014; http://mus.utu.fi/ZMUT.2639.

***Non-type material*** (only diagnostic characters checked): • 23 females; Malaise traps; http://mus.utu.fi/ZMUT.16040, http://mus.utu.fi/ZMUT.16045, http://mus.utu.fi/ZMUT.18110, http://mus.utu.fi/ZMUT.14676, http://mus.utu.fi/ZMUT.25515, http://mus.utu.fi/ZMUT.24981, http://mus.utu.fi/ZMUT.15161, http://mus.utu.fi/ZMUT.16183, http://mus.utu.fi/ZMUT.16629, http://mus.utu.fi/ZMUT.18522, http://mus.utu.fi/ZMUT.19713, http://mus.utu.fi/ZMUT.3413, http://mus.utu.fi/ZMUT.14941, http://mus.utu.fi/ZMUT.26556, http://mus.utu.fi/ZMUT.26292, http://mus.utu.fi/ZMUT.26356, http://mus.utu.fi/ZMUT.28678, http://mus.utu.fi/ZMUT.15660, http://mus.utu.fi/ZMUT.27211, http://mus.utu.fi/ZMUT.26415, http://mus.utu.fi/ZMUT.26143, http://mus.utu.fi/ZMUT.25790, http://mus.utu.fi/ZMUT.16684.

##### Diagnosis.

This species can be distinguished from all other species of the genus by the basally uncoloured wings and entirely black metasomal tergites. This species is also distinctly smaller than its only known congeners: *X. oculatus* and *X. schoutedeni*.

##### Description.

**Female**: forewing length about 6.6 mm. ***Head*** polished. Malar space about 0.3× as long as basal mandibular width; mandible base oblique, about 34° from vertical axis of face. Lower face about 1.2× as wide as medially high, bearing very weak setiferous punctures. Flagellum with 21 flagellomeres; F1 about 1.35× as long as F2. Head, in dorsal view, with gena evenly rounded behind eye. Lateral ocellus separated from compound eye by about 1.25× its own maximum diameter; ocelli forming a subequilateral triangle, with distance between anterior and posterior ocellus about 0.7× of distance between posterior ocelli. ***Mesosoma*** polished. Pronotum mostly bare, with very weak epomia. Mesoscutum bare except for short, sparse hairs on margins. Mesopleuron mostly bare, with short, sparse hairs ventrally and near dorsal margin. Metapleuron bare except for short, sparse hairs on margin at posterior side. Propodeum in profile rather evenly and strongly declivous, dorsally bare, laterally bearing very weak setiferous punctures. Hindwing with abscissa of *Cu1* between *M* and *cu-a* about 1.1× as long as *cu-a*. ***Metasoma*** polished. Tergite 1 in profile rather low, evenly declivous on basal 2/3 and rather flat on apical 1/3, smooth on declivous part, otherwise with short, sparse hairs, about 1.4× as long as apically broad. Tergite 2 with weak setiferous punctures on median more or less triangular area, delineated by lateral oblique grooves extending from the anterior margin to about 0.6 length of tergite, and rather weak posterior transverse grooves, setiferous punctures outside median area comparably dense and strong except for on smooth posterior band, about 0.95× as long as apically broad. Tergites 3–5 covered with rather dense and strong setiferous punctures except for on smooth posterior band and on nearly smooth latero-median tubercles. Ovipositor projecting beyond apex of metasoma by about 4.0× length of hindtibia, shaft straight, followed by downcurved apical part; apex of ventral valve bearing about 14 teeth, progressively becoming weaker and more oblique from the apex towards the base, with a disproportionately large gap between the basal and second most basal teeth, approximately twice the distance between the second and third most basal teeth.

***Colour***: head yellow-orange with black mandibular teeth, ocellar area to central occiput and antenna except for yellow apex of apical flagellomere. Mesosoma yellow-orange with stub-like lateral longitudinal carinae on posterior propodeum black; foreleg yellow; midleg blackish with yellow coxa, femur and dorsal trochanter, and trochantellus; hindleg black with yellow-orange basal margin of coxa, dorsal trochantellus and dorso-basal patch on femur; wings uncoloured with forewing infumate distal of blackish pterostigma, encompassing a weakly yellowish apical patch, and hindwing weakly infumate in apical fourth. Metasoma blackish with white membranous areas of sternites.

**Male.** Unknown.

##### Etymology.

This species is named in honour of the first author’s wife, Iida Österman, who has shown interest in her husband’s work on Darwin wasps.

##### Distribution.

Uganda.

##### Biological notes.

The host(s) are unknown but could be wood-boring insects judging by the similarity of the ovipositor to *Dolichomitus*. The specimens were mainly caught in primary or to a lesser extent disturbed forest, with few caught in clearcut former plantations and none outside the forest in farmland. However, the sample sizes are too low to tell if this apparent preference for undisturbed forest is a genuine pattern (manuscript in prep). Considerably more were caught during the dry seasons than the wet seasons (22 versus 7 specimens), which is a very common pattern in the (Malaise-trapped) Ugandan material.

#### ﻿ Xanthephialtes oculatus

Taxon classificationAnimaliaHymenopteraIchneumonidae

(Brullé, 1846)

09C367A7-D86B-5C2A-A8EC-39D1BE37CA9C

Figs 6–10, 11–16

Ephialtes oculatus Brullé, 1846: 81. Original description and combination.
Xanthephialtes oculatus ; Cameron 1906: 118. New combination (indirect).

##### Holotype.

In Muséum national d’Histoire naturelle, Paris (MNHN).

##### Type locality.

“Afrique Australe” [South Africa].

##### Material examined.

***Holotype*** • 1 female. South Africa; presumably collected sometime during 1818–1820; Pierre Antoine Delalande leg.; MNHN. ***Non-type*** • 1 female. South Africa; SAM-HYM-P000047; SAMC.

##### Distribution.

Burundi, South Africa.

**Figures 6–10.**
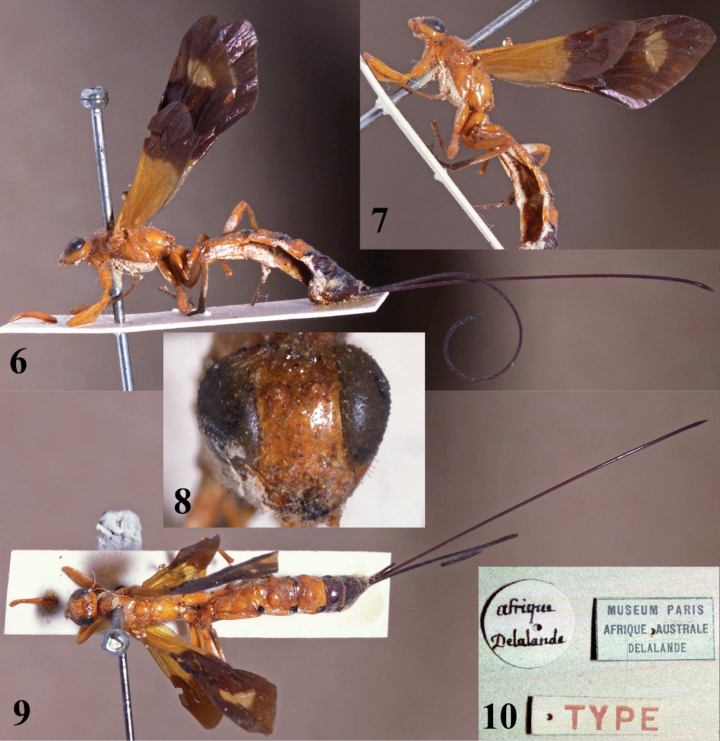
*Xanthephialtes oculatus*, holotype female (MNHN). **6.** Habitus, lateral view; **7.** Body, wings, lateral view; **8.** Head, anterior view; **9.** Habitus, dorsal view; **10.** Data labels.

**Figures 11–16.**
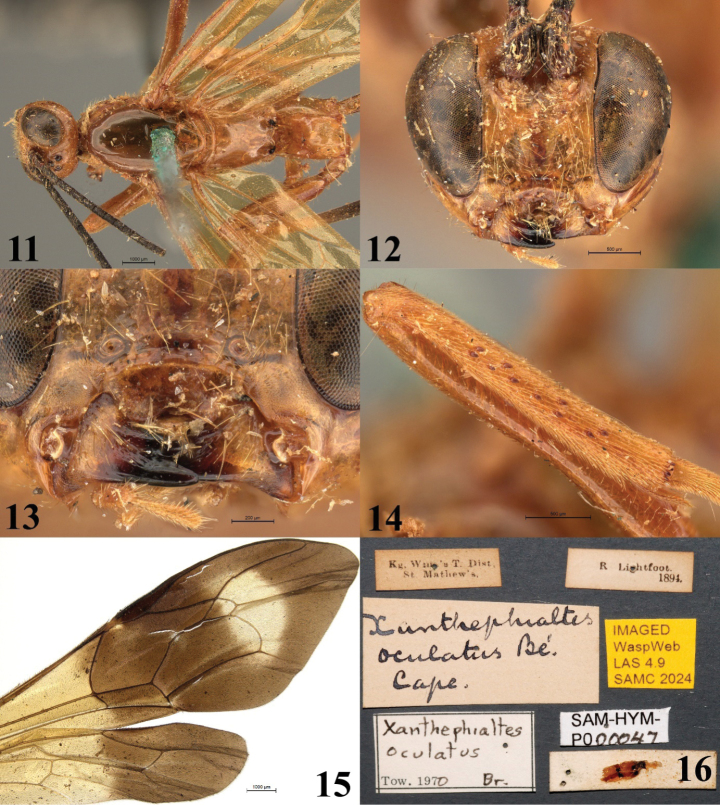
*Xanthephialtes oculatus*, non-type female (SAMC). **11.** Head, mesosoma, dorsal view; **12.** Head, anterior view; **13.** Mandibles; **14.** Flattened bristles on foretibia, antiaxial view; **15.** Wings, dorsal view; **16.** Data labels.

##### Biological notes.

The host(s) and other biological information are unknown. The hosts could be wood-boring insects judging by the similarity of the ovipositor to *Dolichomitus*.

#### ﻿ Xanthephialtes schoutedeni

Taxon classificationAnimaliaHymenopteraIchneumonidae

Benoit, 1954

C7359827-DB93-5437-B92A-85E89FE546F4

Figs 17–21, 22

##### Holotype.

In Musée Royal de l’Afrique Centrale, Tervuren (RMCA).

##### Type locality.

Tshikapa, Democratic Republic of Congo.

##### Material examined.

***Holotype*** • 1 female. Democratic Republic of Congo, Tshikapa; Oct. 1921; Henri Schouteden leg.; RMCA. ***Non-type material*** • 41 females. Uganda, Kibale National Park, Kanyawara; Tapani Hopkins leg.; malaise traps; ZMUT; http://mus.utu.fi/ZMUT.1778, http://mus.utu.fi/ZMUT.2017, http://mus.utu.fi/ZMUT.3951, http://mus.utu.fi/ZMUT.4429, http://mus.utu.fi/ZMUT.6018, http://mus.utu.fi/ZMUT.8590, http://mus.utu.fi/ZMUT.8850, http://mus.utu.fi/ZMUT.11504, http://mus.utu.fi/ZMUT.14921, http://mus.utu.fi/ZMUT.14938, http://mus.utu.fi/ZMUT.15760, http://mus.utu.fi/ZMUT.15931, http://mus.utu.fi/ZMUT.15932, http://mus.utu.fi/ZMUT.15935, http://mus.utu.fi/ZMUT.16020, http://mus.utu.fi/ZMUT.16034, http://mus.utu.fi/ZMUT.16039, http://mus.utu.fi/ZMUT.16143, http://mus.utu.fi/ZMUT.16929, http://mus.utu.fi/ZMUT.17203, http://mus.utu.fi/ZMUT.17249, http://mus.utu.fi/ZMUT.17372, http://mus.utu.fi/ZMUT.17469, http://mus.utu.fi/ZMUT.17501, http://mus.utu.fi/ZMUT.17937, http://mus.utu.fi/ZMUT.18040, http://mus.utu.fi/ZMUT.18056, http://mus.utu.fi/ZMUT.18278, http://mus.utu.fi/ZMUT.19377, http://mus.utu.fi/ZMUT.19378, http://mus.utu.fi/ZMUT.19743, http://mus.utu.fi/ZMUT.19929, http://mus.utu.fi/ZMUT.20593, http://mus.utu.fi/ZMUT.24430, http://mus.utu.fi/ZMUT.24630, http://mus.utu.fi/ZMUT.26240, http://mus.utu.fi/ZMUT.26675, http://mus.utu.fi/ZMUT.26680, http://mus.utu.fi/ZMUT.26895, http://mus.utu.fi/ZMUT.28297, http://mus.utu.fi/ZMUT.28358.

**Figures 17–21.**
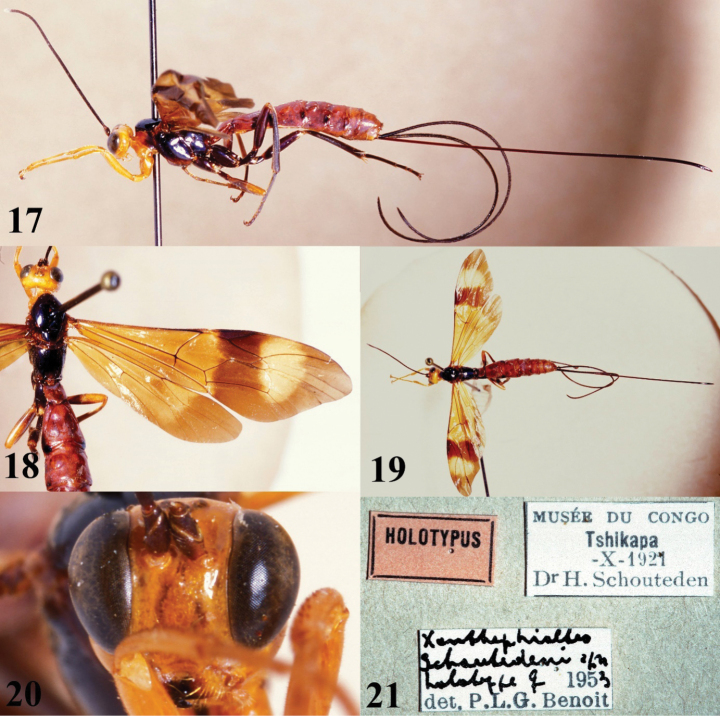
*Xanthephialtes schoutedeni*, Holotype female (RMCA). **17.** Habitus, lateral view; **18.** Head, mesosoma, wings, dorsal view; **19.** Habitus, dorsal view; **20.** Head, anterior view; **21.** Data labels.

##### Distribution.

Democratic Republic of Congo, Guinea, Uganda.

##### Biological notes.

The host(s) are unknown but could be wood-boring insects judging by the similarity of the ovipositor to *Dolichomitus*. The Ugandan specimens were mainly caught in primary forest and seemed to be concentrated around two traps (HILLT1 and R93T2), which were next to the canopies of recently fallen trees. Other traps with dead wood nearby (e.g. CCT1) did not catch noticeably large amounts of specimens.

**Figure 22.**
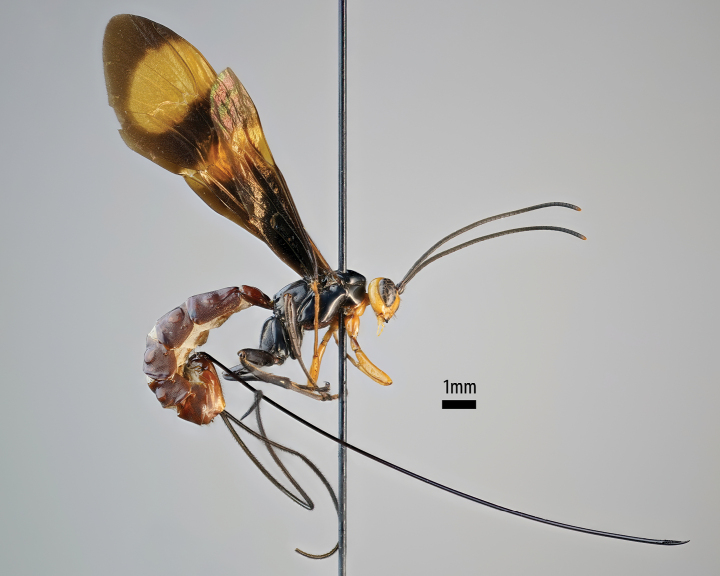
*Xanthephialtes schoutedeni*, non-type female (ZMUThttp://mus.utu.fi/ZMUT.17469); habitus, lateral view.

##### Variation.

The Ugandan specimens differ significantly in colouration compared to the holotype (see Discussion).

## ﻿Discussion

Unfortunately, information on host use is not available for any species of *Xanthephialtes*. We suspect that species of *Xanthephialtes* may, like many other ephialtines with long ovipositors, be idiobiont ectoparasitoids of wood-boring holometabolan insects. Ecological analyses of the Ugandan samples (in prep.) tentatively suggest that both *X. schoutedeni* and *X. iida* are associated with undisturbed forest rather than farmland, and *X. schoutedeni* were caught in much larger numbers in two traps near recently fallen trees (HILLT1 and R93T2). However, otherwise there was no strong association with dead wood for either *X. schoutedeni* or *X. iida*, and the sample sizes are mostly too few for statistically significant results. The shorter ovipositor (on an absolute scale) of *X. iida* suggests it does not oviposit as deep as its congeners, even though the substrate is probably the same.

All 73 *Xanthephialtes* wasps examined here are female. This is an extremely skewed sex ratio for at least *X. iida* and *X. schoutedeni* (we only examined two *X. oculatus*). The likeliest explanation is that the males swarm around a few female emergence sites, and no such swarm sites happen to have been sampled. Male swarming is common in parasitoids of wood-boring hosts (cf. Eggleton 1991; Keronen et al. 2021), and other such species in the Ugandan material have shown similar skewed sex ratios (e.g. the rhyssine *Epirhyssa uelensis* with 158 females and 2 males; Hopkins et al. 2019c).

In addition to the 29 specimens of *X. iida*, the Ugandan samples contained 41 specimens which we have identified as *X. schoutedeni*. The Ugandan specimens differ in colouration, particularly in the wings, compared to the holotype from Tshikapa, Democratic Republic of Congo (Figs 17–19 vs Fig. 22). Most notably, the Ugandan specimens have basally black wings, whereas the holotype has basally yellowish wings with weakly developed blackish areas near the centre of fore- and hindwings, indicating weak development of the Ugandan colour pattern (note how the wings being stacked in Fig. 22 makes them appear even darker). Two other differences between the Ugandan specimens and the holotype are evident: the apical yellow patch on the forewing is larger and the metasoma is more brownish (vs reddish) in the Ugandan specimens. The previously known distribution of *X. schoutedeni* (the Democratic Republic of Congo, and Guinea) suggests it is widespread in Africa and, therefore, is expected to occur in Uganda. Although, with the deterioration of the connection between Kibale and the forest of the Congo Basin (Hartter 2007), rapid allopatric speciation may be ongoing (see more below). For now, in the absence of DNA samples, we interpret these differences as exceptionally strong intraspecific variation.

**Figure 23.**
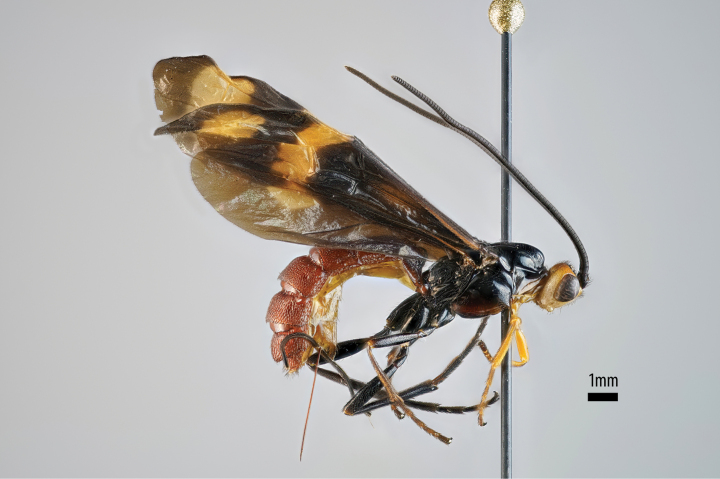
*Mesobracon* sp. (ZMUThttp://mus.utu.fi/ZMUT.2345); habitus, lateral view.

The darker colouration of the Ugandan specimens of *X. schoutedeni* may be explained, for example, by its involvement in a local mimicry ring that favours darker forms. Namely, in the Ugandan samples its colouration (roughly: head and forelegs yellow, mesosoma and remaining legs black, forewing black with a medial and preapical yellow patch, hindwing black with a preapical yellow patch, metasoma reddish) is matched in Pimplinae by a few species of *Camptotypus* Kriechbaumer, 1889 and *Xanthophenax* Saussure, 1892 and in Braconidae: Braconinae by at least one species of *Mesobracon* Szépligeti, 1902 (Fig. 23) and one species from another unidentified genus. Mimicry rings in tropical parasitoid wasps can be taxonomically broad, complex and involve overlapping Batesian and Müllerian mimicry (Porter 1978; Quicke 2015). One possibility is that these ichneumonids are Batesian mimics of Braconinae species with strong chemical defenses, which in turn may mimic each other through Müllerian mimicry; alternatively, all these may mimic aculeate wasps. Establishing the structure of mimicry rings in insects is difficult, however, so this remains speculative.

### ﻿Key to species of *Xanthephialtes* Cameron, 1906

**Table:** 

1	Wings basally uncoloured (A); metasomal tergites entirely black (B)	***Xanthephialtes iida* sp. nov.**
	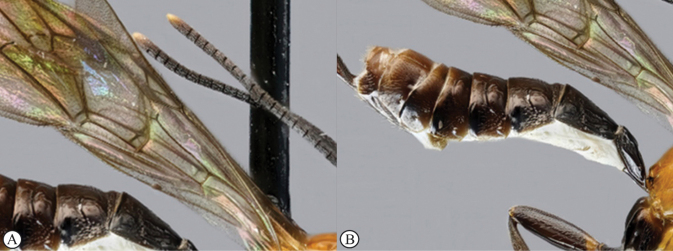	
–	Wings basally yellowish (a) or blackish (b); metasomal tergites not entirely black (c)	**2**
	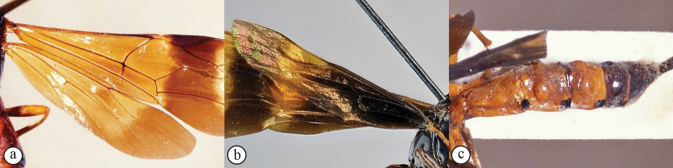	
2	Mesosoma entirely orange (A); metasomal tergites orange with black tergites 5+ and apico-lateral spots on mid tergites (B); wings basally yellowish (C)	***Xanthephialtes oculatus* (Brullé, 1846)**
	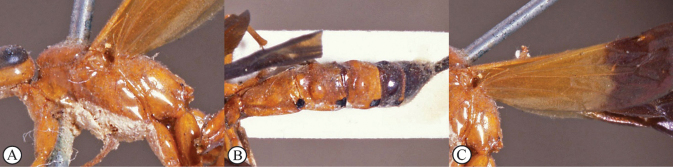	
–	Mesosoma mostly black (a, b); metasomal tergites brownish (a) or reddish with black apico-lateral spots on mid tergites (b); wings basally yellowish (c) or blackish (d)	***Xanthephialtes schoutedeni* Benoit, 1954**
	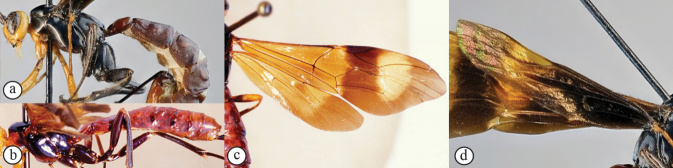	

## Supplementary Material

XML Treatment for Xanthephialtes

XML Treatment for Xanthephialtes iida

XML Treatment for Xanthephialtes oculatus

XML Treatment for Xanthephialtes schoutedeni

## ﻿Additional information

### Use of AI

No use of AI was reported.

### Author ORCIDs

Emil M. Österman https://orcid.org/0009-0008-8307-5804

Tapani Hopkins https://orcid.org/0000-0002-2256-0098

Ilari E. Sääksjärvi https://orcid.org/0000-0002-8107-5607

Kari M. Kaunisto https://orcid.org/0000-0001-6665-0047

Simon van Noort https://orcid.org/0000-0001-6930-9741

### Data availability

All images presented in this paper, as well as supplementary images, are available on WaspWeb at https://www.waspweb.org. The data of the Ugandan wasps is in the Zenodo repository (Österman et al. 2025b, https://doi.org/10.5281/zenodo.17379158).
